# OA13.04. Physical health benefits of health Qigong and Energize programs in American elementary school classrooms

**DOI:** 10.1186/1472-6882-12-S1-O52

**Published:** 2012-06-12

**Authors:** C Wang, D Seo, R Geib, N Wroblewski, M Van Puymbroeck, L Kolbe

**Affiliations:** 1 Indiana University Bloomington, Bloomington, USA; 2 Indiana University School of Medicine, Terre Haute, USA; 3 Monroe County YMCA, Bloomington, USA

## Purpose

With the increasing use of complementary and alternative medicine, mind-body exercises (i.e., Tai Chi, Yoga, and Qigong) have become more popular in the United States. In particular, numerous recent investigations have suggested the positive benefits of Qigong for cardiovascular fitness, musculoskeletal conditions, and stress. However, such research is largely limited to adults and the elderly. Few studies have explored the benefits of Qigong in the pediatric population. Thus, the purpose of this study is to investigate: (1) whether Health Qigong is effective, and (2) how effective it is compared with conventional exercise among elementary school children.

## Methods

A pre- and post-test quasi-experimental design was used to assess the effects of three different modes of exercise: (1) aerobic exercise (Energize), (2) mind-body exercise (Health Qigong), and (3) conventional physical education (PE) activities, in terms of improving health during a 16-week intervention, as measured by Heart Rate (HR), Sit-and-Reach (SR), and Body Mass Index (BMI) in children.

## Results

One hundred and five children provided valid data from two elementary schools in Southern Indiana. Of the 105 students, 57 (35.2%) were boys. The average age was 9 years old. The repeated measures analyses of variance revealed a significant decrease in HR (F=70.54, p<.001, η2 =.409), SR (F=11.68, p<.001, η2 =.103), and BMI (F=41.97, p<.001, η2 =.292). In particular, BMI decreased more quickly from the Health Qigong group, with a mean change of 0.698 (p<.001), than the Energize (0.197, p<.05) and the PE group (0.224, p<.05).

## Conclusion

Health Qigong can be as effective as aerobic exercise and physical education activities in reducing HR and increasing SR among elementary school children. Given the significant reduction in BMI, Health Qigong should be further investigated on a possible mechanism to help lose body weight.

